# Risk-adjustment methods for all-payer comparative performance reporting in Vermont

**DOI:** 10.1186/s12913-017-2010-0

**Published:** 2017-01-19

**Authors:** Karl Finison, MaryKate Mohlman, Craig Jones, Melanie Pinette, David Jorgenson, Amy Kinner, Tim Tremblay, Daniel Gottlieb

**Affiliations:** 1 Onpoint Health Data, 254 Commercial Street, Suite 257, Portland, ME 04101 USA; 2 Vermont Blueprint for Health, 280 State Dr. Waterbury, Vermont, 05671 USA; 3 U.S. Department Health and Human Services, Vermont Blueprint for Health. Office of the National Coordinator for Health Information Technology, 330 C Street, SW; Floor 7, Washington, DC, 20024 USA; 4 The Dartmouth Institute for Health Policy and Clinical Practice, Dartmouth Hitchcock Medical Center, Lebanon, NH 03766 USA

**Keywords:** Primary care practice, Risk adjustment, APCD, Population health, Accountable care

## Abstract

**Background:**

As the emphasis in health reform shifts to value-based payments, especially through multi-payer initiatives supported by the U.S. Center for Medicare & Medicaid Innovation, and with the increasing availability of statewide all-payer claims databases, the need for an all-payer, “whole-population” approach to facilitate the reporting of utilization, cost, and quality measures has grown. However, given the disparities between the different populations served by Medicare, Medicaid, and commercial payers, risk-adjustment methods for addressing these differences in a single measure have been a challenge.

**Methods:**

This study evaluated different levels of risk adjustment for primary care practice populations – from basic adjustments for age and gender to a more comprehensive “full model” risk-adjustment method that included additional demographic, payer, and health status factors. It applied risk adjustment to populations attributed to patient-centered medical homes (283,153 adult patients and 78,162 pediatric patients) in the state of Vermont that are part of the Blueprint for Health program. Risk-adjusted expenditure and utilization outcomes for calendar year 2014 were reported in 102 adult and 56 pediatric primary-care comparative practice profiles.

**Results:**

Using total expenditures as the dependent variable for the adult population, the r^2^ for the model adjusted for age and gender was 0.028. It increased to 0.265 with the additional adjustment for 3M Clinical Risk Groups and to 0.293 with the full model. For the adult population at the practice level, the no-adjustment model had the highest variation as measured by the coefficient of variation (18.5) compared to the age and gender model (14.8); the age, gender, and CRG model (13.0); and the full model (11.7). Similar results were found for the pediatric population practices.

**Conclusions:**

Results indicate that more comprehensive risk-adjustment models are effective for comparing cost, utilization, and quality measures across multi-payer populations. Such evaluations will become more important for practices, many of which do not distinguish their patients by payer type, and for the implementation of incentive-based or alternative payment systems that depend on “whole-population” outcomes. In Vermont, providers, accountable care organizations, policymakers, and consumers have used Blueprint profiles to identify priorities and opportunities for improving care in their communities.

## Background

In order to achieve the triple aim of improving health outcomes across a population, improving the experience of care, and reducing health care costs, [1] the U.S. Center for Medicare & Medicaid Innovation (CMMI) at the Centers for Medicare and Medicaid Services (CMS) is implementing value-based Advanced Payment Models that reward health outcomes rather than volume of services. The expansion of these models offered through accountable care organizations (ACOs) increasingly emphasizes multi-payer involvement, primary care focused on comprehensive patient-centered care, accountability for outcomes of a whole population, and use of population-level data for care management, comparative evaluation, and performance-based payments [2, 3].

However, in the United States, a complex web of health insurers has developed over time. These include Medicare, which covers individuals over the age of 65 as well as disabled people under 65; Medicaid, which covers lower income individuals and families; and commercial insurers, which cover insured people under 65 and their families. This system has complicated analysis as data are often stored in isolation and contain variation in quality, services, and prices. Some of these challenges can be overcome through development of all-payer claims databases (APCDs), which facilitate a higher level of uniformity. Additionally, CMMI has awarded State Innovation Model (SIM) grants to support investment in health data infrastructure that facilitates data aggregation and consistent measurement across different payers and settings, including practices, hospitals, health service areas (HSAs), and ACOs [4, 5]. However, analytic methods that evaluate “whole-population” outcomes while accounting for diversity among subgroups are needed to meet the demands of Advanced Payment Models.

First, these methods require access to multi-payer data. Efforts to develop statewide APCDs began more than a decade ago and have expanded significantly in recent years. Currently, 18 states have existing APCDs or are in the process of implementing these critical resources. Additionally, 22 states have expressed strong interest in APCDs, and four have voluntary databases; only six states have no APCD-related activity [6]. Sixteen states have legislation enabling collection of claims data [7]. APCDs typically collect eligibility and all-setting claims and pharmacy data for residents of the state from commercial payers and often Medicare and Medicaid data sources. The data are used to generate a wide variety of cost, utilization, and quality measures.

Second, these methods need to support a “whole-population” measurement system that reports on the whole population while adequately adjusting for differences across practices and across their patient populations. With increased emphasis on primary care transformation as foundational for an accountable health system and with the expanded availability of all-payer data, practices and state initiatives need to understand how practice transformation is affecting care and patient health.

Provider and primary care practice profiles are tools often used to assess a provider’s or practice’s performance, and a variety of efforts have been made to address issues related to risk adjustment, measures standardization, and comparability across practices [8–13]. In addition, guidelines for profiling identified by organizations representing physicians, [14, 15] highlight the importance of providing consistent measurement and information across an entire practice population instead of single-payer reports that cover only a portion of the practice’s population and use varying measures standards and methods of risk adjustment.

The state of Vermont has tackled many of the challenges of building an accountable health system. Its Blueprint for Health (Blueprint) program is charged with reforming health care delivery through supporting practices as they transform to patient-centered medical homes (PCMHs), supporting multi-disciplinary community health teams bridge medical and social services, implementing per patient per month payment reforms, and using data to drive quality improvement at multiple levels – practice, organization, HSA, and state [16]. With access to the state’s APCD, which includes expenditure, utilization, and quality data from commercial, Medicaid, and Medicare payers, as well as clinical data from electronic medical records, the Blueprint has developed practice profiles for each PCMH’s “whole population” of patients. These profiles use a single measurement system with large sample sizes and a consistent method of measures generation, risk adjustment, and measurement of error in estimates. The result is a profile that evaluates a medical home’s whole patient population through claims and clinical data and compares outcomes to other medical homes, HSAs, and the state.

This study describes the creation of these provider-specific reports. Specifically, it describes the risk adjustment and other methods developed to evaluate whole populations. Beyond providing meaningful information to the practices, this study, in achieving its aim, also contributes to the ongoing discussion of how to assess the effect of alternative payment methods on realizing an accountable health system and the Triple Aim.

## Methods

### Data sources

The data used to generate cost, utilization, and quality measures and risk-adjustment methods for Blueprint practice profiles includes membership and claims data reported to the state’s APCD, the Vermont Health Care Uniform Reporting & Evaluation System (VHCURES). The data contained services incurred during calendar year 2014 by Vermont residents enrolled in commercial health plans, Medicaid enrollees for whom Medicaid was the primary payer, and Medicare fee-for-service beneficiaries for whom Medicare was the primary payer. Each member was attributed to a Blueprint primary care practice using a consistent cross-payer methodology that used the plurality of primary care visits based on Evaluation and Management (E&M) codes over a 24-month look-back period. Because patients may have visited different practitioners within the same primary care practice group, attribution was made at the practice level. The APCD data was used to generate a wide range of claims-based cost, utilization, and quality measures. Each practice was compared to all Blueprint practices in the practice’s geographic (HSA) and to all practices across the state. Practice-level data is not publically available but practice data rolled-up to HSA level is publically reported [17].

### Methods of risk adjustment

#### Stratification of pediatric & adult populations

Two types of primary care practice measure sets and profiles were generated: adult (ages 18 years and older) and pediatric (ages 1–17 years). The adult profiles included members with commercial, Medicaid (ages 18–64 years), or Medicare (ages 18 years and older) as primary payer. The pediatric profiles included members ages 1–17 years with commercial payers or Medicaid as primary.

While many practices treat both adult and pediatric populations, others treat primarily only adults or children. Because the pediatric and adult populations have very different health, utilization, and cost distributions, segregating profiles by adult and pediatric populations provides a more accurate look at practice differences. An alternative — basing practice profiles on physician specialty — would be problematic since attribution is at the practice level and practice groups may have included practitioners with different primary care specialties.

Newborn infants under the age of 1 year were not included since they (1) have high cost compared to the remainder of the pediatric population, (2) have a small number of outlier cases requiring neonatal intensive care, and (3) are often reported as bundled newborn claims by payers resulting in incomplete reporting of expenditures.

#### Treatment of outliers

The method used in this study capped outliers in expenditure and utilization at the 99th percentile of patients for each measure. Capping was done at the state-level for each major payer type (i.e., commercial, Medicaid, Medicare), and capped values were used for practice-level analysis. For the 2014 study population, the dollars truncated by capping represented 7% for the adult population and 13% for the pediatric population.

#### Adjustment for demographics & health status

Demographic and health status information determined from the APCD data formed the basis for the risk-adjustment methods used for the Blueprint Practice Profiles. These factors included age, gender, presence of a Blueprint-selected chronic condition, health status as measured by 3M Clinical Risk Groups (CRGs), and (for adult profiles) the occurrence of a maternity diagnosis.

Adjustments were made for the partial length of enrollment in payer insurance reported for some members during the calendar year. Average members — i.e., cumulative member months divided by 12 — were reported for each practice.

For the purposes of risk adjustment and to facilitate interpretation of results, member age was grouped. Due to the potential for interaction effects of age and gender, the full model used age and gender groupings (e.g., males aged 18–34 years, females aged 18–34, etc.).

There are several systems for measuring health status being used in the United States, each with its different point of emphasis, yet no single system has emerged as the “gold standard.” In this study, the primary method of adjustment for each member’s health status was based on the application of 3M Clinical Risk Groups (CRGs) to the APCD data. CRGs used to measure health status are applicable to all ages, are updated regularly, are designed for use with claims data, are transparent in documentation, perform as well as other available systems, and represent a practical solution to meet the needs of the Blueprint project [18]. The grouper classifies each member into a hierarchy of 1080 distinct clinical groups and nine major clinical CRG statuses based on the diagnoses reported. Due to small numbers, and to create an efficient model that was easily understood, these nine categories were further combined during the risk model development process into (1) Healthy (reference group), (2) Acute or Minor Chronic (e.g., acute ear, nose, or throat condition or minor join pain), (3) Moderate Chronic (e.g., diabetes or moderate chronic joint pain), (4) Significant Chronic (e.g., diabetes with other comorbid conditions such as congestive heart failure (CHF) or chronic obstructive pulmonary disease (COPD)), and (5) Cancer or Catastrophic (e.g., malignant breast cancer, HIV, cystic fibrosis, muscular dystrophy, quadriplegia).

The Blueprint program also targeted select chronic conditions: asthma, chronic obstructive pulmonary disorder (COPD), congestive heart failure (CHF), depression, diabetes, hypertension, ischemic heart disease, and attention deficit disorder (pediatric model only). A “chronic” (0/1) variable was created if a member was identified with any of these conditions. Since CRGs do not include pregnancy and child birth in clinical classification, a “maternity” (0/1) variable was created for members with pregnancy or delivery claims during the year.

#### Adjustment for practice’s Medicaid & Medicare population

The primary care practice profiles combine the populations from three different payer types (or “payers”) — commercial, Medicaid, and Medicare — that have significant differences in demographics, socioeconomic status, health status, provider reimbursement structures, and services covered and used. For the full model, Medicaid members were identified in the indicator variable as: Commercial = 0, Medicare = 0, Medicaid = 1.

Another distinguishing attribute of the Medicaid data was the inclusion of members who received “special Medicaid services” (SMS) uncommon in the commercial and Medicare populations. Members receiving SMS may have had a level of disability not adjusted for through the CRGs. Examples of SMS include members receiving day treatment, residential treatment, case management services, and special school services covered by the Department of Education. These types of services can contribute significantly to a member’s total expenditures. After evaluation of statistical distributions for these services, members with SMS expenditures over $500 during the 12-month study period were identified by a binomial (0/1) variable.

During model development, it was determined that a practice’s percentage of total members covered by Medicaid was a statistically significant predictor of higher total expenditures. Practices in Vermont varied considerably in their percentage of members who were covered by Medicaid. Therefore, the full risk-adjustment model included a practice’s percent Medicaid for each Medicaid enrollee in the practice.

Given widely observed healthcare disparities, women covered by Medicaid may be at higher risk for poor maternity and neonatal outcomes than women covered by commercial plans [19–22]. To account for these differences, an interaction term was added between Medicaid and maternity.

As was done for Medicaid, the full risk-adjustment model identified Medicare-eligible beneficiaries through the indicator variable: Commercial = 0, Medicaid = 0, Medicare = 1. The model also included a variable for “practice’s percent Medicare” for members contributing to the practice’s percent Medicare. Using Medicare-specific eligibility elements, “disability” (0/1) and “end-stage renal disease” (0/1) variables also were created. Pediatric members covered by Medicare were excluded from the pediatric profiling due to small numbers.

#### Full model & the computation of risk-adjusted rates

The risk-adjustment methods used for reporting used SAS (Version 9.3) regression methods (SAS GENMOD procedure). The full model included age/gender stratification groups, Blueprint-selected chronic conditions, CRG classification, maternity status, and the additional Medicaid and Medicare adjustors. Adjusted rates were produced by summing the differences between each member’s actual value and the member’s predicted measurement from the model. Rates were weighted for partial lengths of enrollment. Detailed descriptions of the model’s computation of risk-adjusted rates and 95% confidence intervals for the adult and pediatric populations are provided below.

To calculate the adjusted rate, adjusted values were computed for each member by adding model residuals (e) to the population grand mean $\left(\overline{y}\right)$. To report the overall adjusted rate for each practice, the mean of the adjusted values for the members in each practice $\left({\overline{y}}_{\mathrm{practice}}\right)$, in each HSA ($\overline{y}$_hsa_), and statewide ($\overline{y}$_statewide_) were computed. The following equations represent the models for the adult and pediatric practice profiles.

#### Adult model

$$ \begin{array}{l}y = \alpha + \left(F\_ AGE1834\right){\beta}_1 + \left(F\_ AGE3544\right){\beta}_2 + \left(F\_ AGE4554\right){\beta}_3 + \left(F\_ AGE5564\right){\beta}_4 + \\ {}\left(F\_ AGE6574\right){\beta}_5+\left(F\_ AGE7584\right){\beta}_6+\left(F\_ AGE85 PLUS\right){\beta}_7 + \left(M\_ AGE3544\right){\beta}_8 + \\ {}\left(M\_ AGE4554\right){\beta}_9 + \left(M\_ AGE5564\right){\beta}_{10} + \left(M\_ AGE6574\right){\beta}_{11}+\left(M\_ AGE7584\right){\beta}_{12}+\\ {}\left(M\_ AGE85 PLUS\right){\beta}_{13} + (MEDICAID){\beta}_{14} + (MEDICARE){\beta}_{15} + \left( DUAL\  ELIGIBILITY\right){\beta}_{16}+\\ {}\ (SMS){\beta}_{17} + \left( PRACTICE\_ PERCENT\_ MEDI\right){\beta}_{18}+\left( PRACTICE\_ PERCENT\_ MCARE\right){\beta}_{19} + \\ {}(DISABLED){\beta}_{20} + (ESRD){\beta}_{21}+(CHRONIC){\beta}_{22} + \left(CRG\_ ACUTE\_ MINOR\right){\beta}_{23} + \\ {}\left(CRG\_ CHRONIC\right){\beta}_{24} + \left(CRG\_ SIGNIFICANT\_ CHRONIC\right){\beta}_{25} + \\ {}\left(CRG\_ CANCER\_ CATASTROPHIC\right){\beta}_{26} + (MATERNITY){\beta}_{27} + \left( MATERNITY*\  MEDICAID\right){\beta}_{28} + \varepsilon \end{array} $$

#### Pediatric model

$$ \begin{array}{l}y = \alpha + \left(F\_ AGE0104\right){\beta}_1 + \left(M\_ AGE0511\right){\beta}_2 + \left(F\_ AGE0511\right){\beta}_3 + \left(F\_ AGE1217\right){\beta}_4 + \\ {}\left(M\_ AGE1217\right){\beta}_5 + (MEDICAID){\beta}_6 + (SMS){\beta}_7 + \left( PRACTICE\_ PERCENT\_ MEDI\right){\beta}_8+\\ {}\left( CHRONIC\_ PED\right){\beta}_9 + \left(CRG\_ ACUTE\_ MINOR\right){\beta}_{10} + \left(CRG\_ CHRONIC\right){\beta}_{11} + \\ {}\left(CRG\_ SIGNIFICANT\_ CHRONIC\right){\beta}_{12} + \left(CRG\_ CANCER\_ CATASTROPHIC\right){\beta}_{13} + \varepsilon \end{array} $$

$$ \overline{y} = \left(\frac{{\displaystyle \sum }{y}_i}{MMA}\right) $$

$$ {y}_{\mathrm{adj}}=\overline{y}+e $$

$$ e=y-\widehat{y} $$

$$ {\overline{y}}_{\mathrm{practice}} = \left(\frac{{\displaystyle \sum }{y}_{ad{j}_i}}{{\displaystyle \sum }MM{A}_i}\right)\;\mathrm{f}\mathrm{o}\mathrm{r}\ \mathrm{each}\ \mathrm{practice} $$

$$ {\overline{y}}_{\mathrm{hsa}} = \left(\frac{{\displaystyle \sum }{y}_{ad{j}_i}}{{\displaystyle \sum }MM{A}_i}\right)\;\mathrm{f}\mathrm{o}\mathrm{r}\ \mathrm{the}\ \mathrm{practices}\ \mathrm{in}\ \mathrm{each}\ \mathrm{H}\mathrm{S}\mathrm{A} $$

$$ {\overline{y}}_{\mathrm{statewide}} = \left(\frac{{\displaystyle \sum }{y}_{ad{j}_i}}{{\displaystyle \sum }MM{A}_i}\right)\;\mathrm{f}\mathrm{o}\mathrm{r}\ \mathrm{all}\ \mathrm{members}\ \left(\mathrm{equals}\ \mathrm{the}\ \mathrm{grand}\ \mathrm{mean}\right) $$

where:

- *α* is the intercept
- *ε* is the error term
- *ŷ* is the predicted value from the regression model for each member
- *e* is the residual
- *MMA* is the average enrollment for each participant (i.e., the cumulative member months of enrollment during the year divided by 12)
- Subscript _*i*_ indicates a value for an individual member

For practice-level reporting, 95% confidence intervals were generated based on the standard error of the mean. For utilization measures (e.g., inpatient hospitalizations), the Poisson distribution was utilized. The outlier capping and risk-adjustment models were run separately for each individual expenditure and utilization measure reported to practices.

#### Outcome measures

Blueprint practice profiles reported included 27 expenditure, 15 utilization, 10 HEDIS, and 5 additional National Quality Forum (NQF) endorsed measures. This study focused on four measures for the adult and pediatric practice reporting: (1) total expenditures per capita, (2) total expenditures per capita excluding SMS, (3) total Resource Use Index excluding SMS, and (4) a quality composite measure constructed from HEDIS measures (described below). Total expenditures were based on the allowed amount on claims, which includes both the plan payments and the member’s out-of-pocket payments (i.e., deductible, coinsurance, and copayment). For enhanced expenditure parity across payers, an additional total expenditures measure — this time excluding SMS costs — was also examined. Within the Medicaid population, SMS represented 26% of adult Medicaid population expenditures and 61% of pediatric Medicaid population expenditures.

Because pricing and reimbursement can vary, total expenditure measures do not provide a measure of actual consumption of resources (i.e., the actual frequency and intensity of all services used). Therefore, Blueprint used a measure of overall resource use: the total Resource Use Index (RUI), which is based on the NQF-endorsed measure (NQF #1598) Total Care Relative Resource Values (TCRRVs). The measure was implemented by applying HealthPartners’ Total Cost of Care (TCOC) software to the claims data [23]. The resulting standardized relative RUI was included to measure aggregate resource consumption across all components of care (i.e., inpatient, outpatient facility, professional, and pharmacy). The RUI for each practice was calculated by dividing the adjusted TCRRV rate by the statewide TCRRV rate. Since the TCRRVs do not include many of the special Medicaid services, these services were excluded from the RUI.

Effective and preventive care measures were produced by applying NCQA HEDIS specifications to the APCD data. A composite adult measure was constructed at the practice level using the practice average for three HEDIS measures: comprehensive diabetes care hemoglobin A1c testing, breast cancer screening, and imaging studies for low back pain. A composite pediatric measure was constructed using the practice average for three HEDIS measures: well-child visits, appropriate testing for pharyngitis, and appropriate treatment for upper respiratory infection. These measures were a subset of six adult and four pediatric HEDIS measures available in the profile data and were selected based on sufficient sample size at the practice level for statistical reliability and to ensure that no focus area was overweighted (e.g., limiting cancer screening measures to a single measure). Rates for HEDIS measures were not risk adjusted, and NCQA provides no recommendations for risk adjustment.

#### Comparing risk-adjustment models

The following risk-adjustment models were compared:

- No adjustment
- Age and gender (no interaction)
- Age and gender (no interaction) and CRGs
- Full model (includes age, gender, CRGs, maternity, and payer-specific variables)

At the patient level, the percentage of variance explained by each model was evaluated using the regression r^2^. The relative difference between models was evaluated using the log-likelihood ratio test. At the practice level, each model’s results were evaluated using the coefficient of variation (CV).

## Results

During 2014, an average of 283,153 adult patients and 78,162 pediatric patients were attributed to patient-centered medical homes in the Blueprint program. Blueprint delivered 102 adult and 56 pediatric comparative primary care practice profiles to medical homes that met a minimum volume threshold of 300 patients.

For the adult “whole population,” mean total expenditures were $7297, while mean expenditures excluding SMS were $6941. For the pediatric “whole population,” mean total expenditures were $3062, while mean expenditures without SMS were $1599. For the “whole population,” SMS represented 5% of adult expenditures and 52% of pediatric expenditures.

### Practice-level variation in demographic, health status, & payer predictors

Results revealed substantial variation across Blueprint primary care practices regarding payer mix, demographics, and health status of the patient population. Tables 1 and 2 provide descriptive statistics for the adult and pediatric populations including mean and range of practice size, age, and proportions for gender, CRGs, targeted chronic conditions, maternity status (adult only, Table 1), and Medicaid and Medicare status. The results also describe practice-level variation. Based on coefficients of variation, practices in the adult population varied less on age (9.8) and more on patients with maternity (81.6), special Medicaid services (67.0), Medicare dual-eligible status (48.3), and significant chronic conditions CRG (42.4) (in descending order). For the pediatric population, practices varied less on age (8.4) and more on significant chronic conditions CRG (46.9) special Medicaid services (35.2), patients with Medicaid (27.7), and chronic conditions CRG (24.5) (in descending order).

**Table 1:** Vermont Blueprint for Health Practice Variation in Demographics, Health Status, & Payer Mix – Adult Population, CY2014

Metric	Patient-Level Variation (*N* = 283153)	Practice-Level Variation (*N* = 102)							
	Mean	Mean	SD	CV	Median	IQR (25%)	IQR (75%)	Min.	Max.
Practice Size	N/A	2699.7	2010.2	74.5	2233.0	1272	3578	352	10756
Age (in Years)	50.0	52.0	5.1	9.8	51.7	49.1	53.5	40.1	67.7
Gender = Male	45.1%	44.9%	7.1%	15.9	46.1%	42.3%	48.7%	9.3%	58.6%
Clinical Risk Group									
Healthy	Reference group								
Acute/Minor	19.9%	19.7%	2.5%	12.5	19.8%	18.3%	21.1%	13.2%	26.2%
Chronic	24.1%	24.8%	3.9%	15.5	25.0%	22.5%	27.0%	14.5%	35.4%
Significant chronic	12.4%	13.4%	5.7%	42.4	12.5%	9.7%	15.0%	2.1%	36.3%
Cancer or catastrophic	1.4%	1.5%	0.7%	43.5	1.4%	1.2%	1.7%	0.0%	4.3%
Targeted chronic conditions	43.9%	44.8%	8.0%	17.8	44.9%	39.7%	49.9%	17.6%	65.5%
Maternity	1.6%	1.5%	1.3%	81.6	1.3%	1.0%	1.7%	0.0%	10.8%
Medicaid	18.8%	19.0%	9.1%	48.1	18.4%	13.0%	25.7%	1.0%	47.6%
Special Medicaid services	3.8%	3.6%	2.4%	67.0	3.2%	2.2%	4.9%	0.0%	14.9%
Medicare	26.3%	29.2%	11.2%	38.3	28.8%	23.9%	34.3%	0.0%	63.3%
Medicare dual eligible	6.3%	6.8%	3.3%	48.3	6.9%	4.6%	8.9%	0.0%	15.1%
Medicare disabled	6.8%	7.2%	3.1%	43.6	7.0%	5.2%	9.0%	0.0%	15.9%
Medicare End-Stage Renal Disease (ESRD)	0.08%	0.08%	0.09%	109.3	0.06%	0.00%	0.11%	0.00%	0.48%

All percentages use the whole population as denominator and are not specific to payer (e.g., Special Medicaid Services for Medicaid). Examples of special Medicaid services include patients receiving day treatment, residential treatment, case management services, and special school services covered by the Department of Education. Clinical Risk Groups utilize a hierarchy to classify a patient into one and only one CRG for the year. Example conditions include Acute or Minor Chronic (e.g., acute ear, nose or throat condition or minor join pain), Moderate Chronic (e.g., diabetes or moderate chronic joint pain), Significant Chronic (e.g., diabetes with other comorbid conditions such as CHF or COPD), and Cancer or Catastrophic (e.g., malignant breast cancer, HIV, cystic fibrosis, muscular dystrophy, quadriplegia). Target Conditions include asthma, chronic obstructive pulmonary disease, congestive heart failure, ischemic heart disease, hypertension, depression, diabetes, and attention deficit disorder

*SD* Standard deviation, *CV* Coefficient of variation, *IQR* Interquartile range

**Table 2:** Vermont Blueprint for Health Practice Variation in Demographics, Health Status, & Payer Mix – Pediatric Population, CY2014

Metric	Patient Level (*N* = 78162)	Practice-Level Variation (*N* = 56)							
	Mean	Mean	SD	CV	Median	IQR (25%)	IQR (75%)	Min.	Max.
Practice size	NA	1264.7	1109.7	87.7	880.5	455	1678	317	5303
Age (in years)	9.1	9.4	0.8	8.4	9.3	8.7	10.1	7.7	11.1
Gender = Male	51.2%	51.0%	2.4%	4.8	51.1%	49.6%	52.8%	44.9%	55.4%
Clinical risk groups									
Healthy	Reference group								
Acute/Minor	15.8%	16.1%	2.1%	12.7	16.1%	15.0%	17.4%	10.9%	21.0%
Chronic	8.8%	8.6%	2.1%	24.5	8.3%	7.2%	9.6%	5.0%	16.6%
Significant chronic	1.1%	1.0%	0.5%	46.9	1.0%	0.6%	1.3%	0.2%	2.3%
Cancer or catastrophic	0.2%	0.2%	0.1%	92.4	0.1%	0.0%	0.3%	0.0%	0.7%
Targeted chronic conditions	20.0%	20.2%	3.9%	19.3	19.9%	17.5%	22.9%	12.2%	30.3%
Medicaid	53.8%	54.3%	15.0%	27.7	56.6%	43.6%	64.8%	24.8%	82.9%
Special Medicaid services	18.1%	18.3%	6.4%	35.2	18.3%	13.2%	22.0%	6.9%	33.4%

All percentages use the whole population as denominator and are not specific to payer (e.g., Special Medicaid Services for Medicaid). Examples of special Medicaid services include patients receiving day treatment, residential treatment, case management services, and special school services covered by the Department of Education. Clinical Risk Groups utilize a hierarchy to classify a patient into one and only one CRG for the year. Example conditions include Acute or Minor Chronic (e.g., acute ear, nose or throat condition or minor join pain), Moderate Chronic (e.g., diabetes or moderate chronic joint pain), Significant Chronic (e.g., diabetes with other comorbid conditions such as CHF or COPD), and Cancer or Catastrophic (e.g., malignant breast cancer, HIV, cystic fibrosis, muscular dystrophy, quadriplegia). Target Conditions include asthma, chronic obstructive pulmonary disease, congestive heart failure, ischemic heart disease, hypertension, depression, diabetes, and attention deficit disorder

*SD* Standard deviation, *CV* Coefficient of variation, *IQR* Interquartile range

### Comparing risk-adjustment models

Comparative results for each risk-adjustment model are provided in Table 3. The model for total expenditures for the adult population resulted in an r^2^ of 0.028 when adjusted for only age and gender. The r^2^ increased to 0.265 with the addition of CRGs and to 0.293 in the full model. A similar pattern for the r^2^ was found for expenditures excluding SMS and for total resource use excluding SMS. The log-likelihood ratio test indicated that the model differences were statistically significant. For total expenditures at the practice level for the adult population, the no-adjustment model had the highest practice-level variation as measured by the coefficient of variation (18.5) compared to the age and gender model (14.8); the age, gender, and CRG model (13.0); and the full model (11.7). Again, a similar pattern for the CV was found for expenditures excluding SMS and for total resource use excluding SMS.

**Table 3:** Vermont Blueprint for Health Risk-Adjustment Model Comparisons & Resulting Practice Level Coefficient of Variation, CY2014

Model	Adult Model			Pediatric Model		
	r^2^	D = Log-Likelihood Ratio (p-value)	Model Adjusted Practice Level Coefficient of Variation	r^2^	D = Log-Likelihood Ratio (p-value)	Model Adjusted Practice Level Coefficient of Variation
Total expenditures						
No adjustment	N/A		18.5	N/A		23.4
Age and gender	0.028	8102 (*p* < .001)	14.8	0.008	647 (*p* < .001)	24.3
Age, gender, and CRG	0.265	79231 (*p* < .001)	13.0	0.253	22138 (*p* < .001)	19.3
Full model	0.293	10884 (*p* < .001)	11.7	0.352	11092 (*p* < .001)	11.4
Total expenditures excluding SMS						
No adjustment	N/A		19.1	N/A		15.0
Age and gender	0.038	11116 (*p* < .001)	14.2	0.007	524 (*p* < .001)	15.3
Age, gender, and CRG	0.270	78242 (*p* < .001)	14.3	0.288	25982 (*p* < .001)	9.9
Full model	0.285	5779 (*p* < .001)	12.1	0.302	1622 (*p* < .001)	9.3
Total resource use excluding SMS						
No adjustment	N/A		22.4	N/A		17.0
Age and gender	0.045	12904 (*p* < .001)	17.3	0.007	561 (*p* < .001)	17.4
Age, gender, and CRG	0.266	74684 (*p* < .001)	13.6	0.289	26054 (*p* < .001)	11.7
Full model	0.289	8805 (*p* < .001)	11.5	0.315	3003 (*p* < .001)	9.0
HEDIS composite (not adjusted)			5.4			7.8

The r^2^ represents the percentage explained by the model at the patient level. The mode-adjusted practice-level coefficient of variation represents the degree of variability in rates between practices at the practice level, with a lower value indicating reduced variation

The model for total expenditures for the pediatric population resulted in an r^2^ of 0.008 when adjusted for only age and gender. The r^2^ increased to 0.253 with the addition of CRGs and to 0.352 in the full model. A similar pattern was found for expenditures excluding SMS and total resource use excluding SMS. As with the adult comparisons, the log-likelihood ratio test for pediatrics indicated that the model differences were statistically significant. For total expenditures at the practice level for the pediatric population, the no-adjustment model had a higher CV (23.4) compared to the full model (11.4). Again, a similar pattern for the CV was found for expenditures excluding SMS and for total resource use excluding SMS.

The HEDIS composite measures were not risk adjusted. The practice-level coefficient of variation for the adult (5.4) and pediatric (7.8) populations were both lower than variation in the expenditure or RUI measures.

### Primary care practice-level variation & associations

Compared with the no-adjustment model, the full model showed reduced variation at the practice level. For the adult population, the practice-level range in per person annual expenditures without adjustment ($3506–$13,056) was reduced in the full model ($5113–$9666). For the pediatric population, the same trend held true, with the practice-level range in per-person annual expenditures without adjustment ($1112–$2193) demonstrating a reduction in the full model ($1331–$2002).

When stratifying the practices’ average per person annual expenditures excluding SMS into quintiles for both the adult and pediatric populations, the results (Table 4) demonstrated a reduction in the variability between practices in the full model compared to the no-adjustment model. Comparing the first (i.e., the lowest average annual expenditures) and fifth (i.e., the highest average annual expenditures) quintiles, the full model decreased the range by $1335 (37%) for the adult population practices and by $238 (37%) for the pediatric population practices relative to non-risk-adjusted rates. In addition, a large proportion of practices shifted into a different expenditure quintile in the full model compared to no adjustment – 77% for adult population practices and 54% for pediatric population practices.

**Table 4:** Vermont Blueprint for Health Practice-Level Comparison of Mean Total Expenditures Excluding Special Medicaid Services (SMS) – No Adjustment vs. Full Model, CY2014

Total Expenditures Excluding Special Medicaid Services (SMS) –Quintiles	Adult Practices (*N* = 102)		Pediatric Practices (*N* = 56)	
	No-Adjustment Mean	Full-Model Mean	No-Adjustment Mean	Full-Model Mean
1	$5555	$5812	$1301	$1415
2	$6464	$6424	$1454	$1517
3	$7093	$6823	$1578	$1596
4	$7886	$7189	$1738	$1664
5	$9179	$8101	$1949	$1825

For adult practices, compared to the no-adjustment model, the full model changed the quintile for 79 (77%) of the practices, with 41 practices shifting to a higher expenditure quintile and 38 changing to a lower expenditure quintile (23 remained in the same quintile). For pediatric practices, compared to the No-Adjustment Model, the Full Model changed the quintile for 30 (54%) of the practices, with 16 practices moving to a higher expenditure quintile and 14 changing to a lower expenditure quintile (26 remained in the same quintile). Examples of special Medicaid services include patients receiving day treatment, residential treatment, case management services, and special school services covered by the Department of Education

**Fig. 1:**
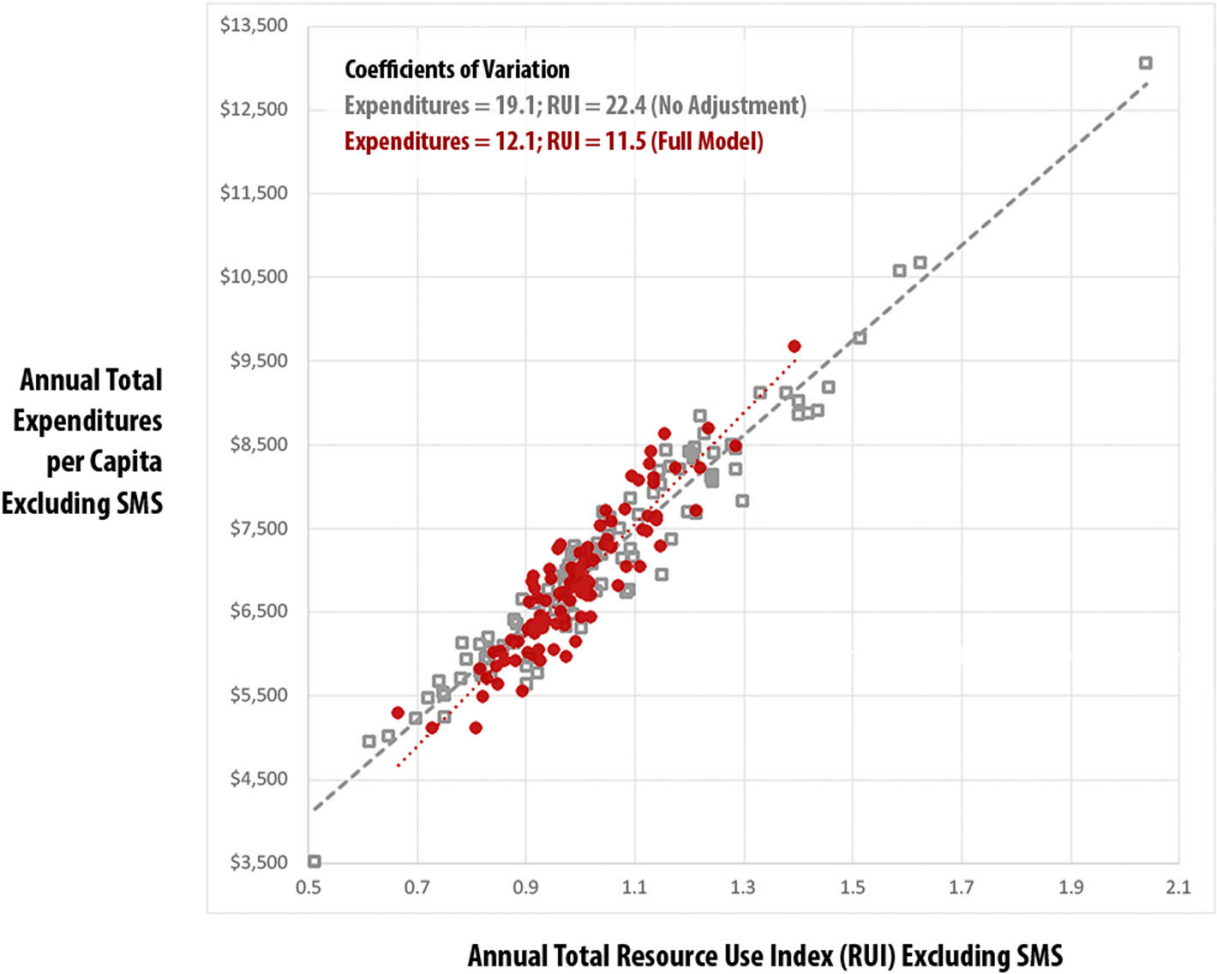
Vermont Blueprint for Health Practice-Level Comparison: Total Expenditures vs. Total Resource Use Index (RUI) Excluding Special Medicaid Services (SMS) – Adult, No Adjustment vs. Full Model, CY2014

**Fig. 2:**
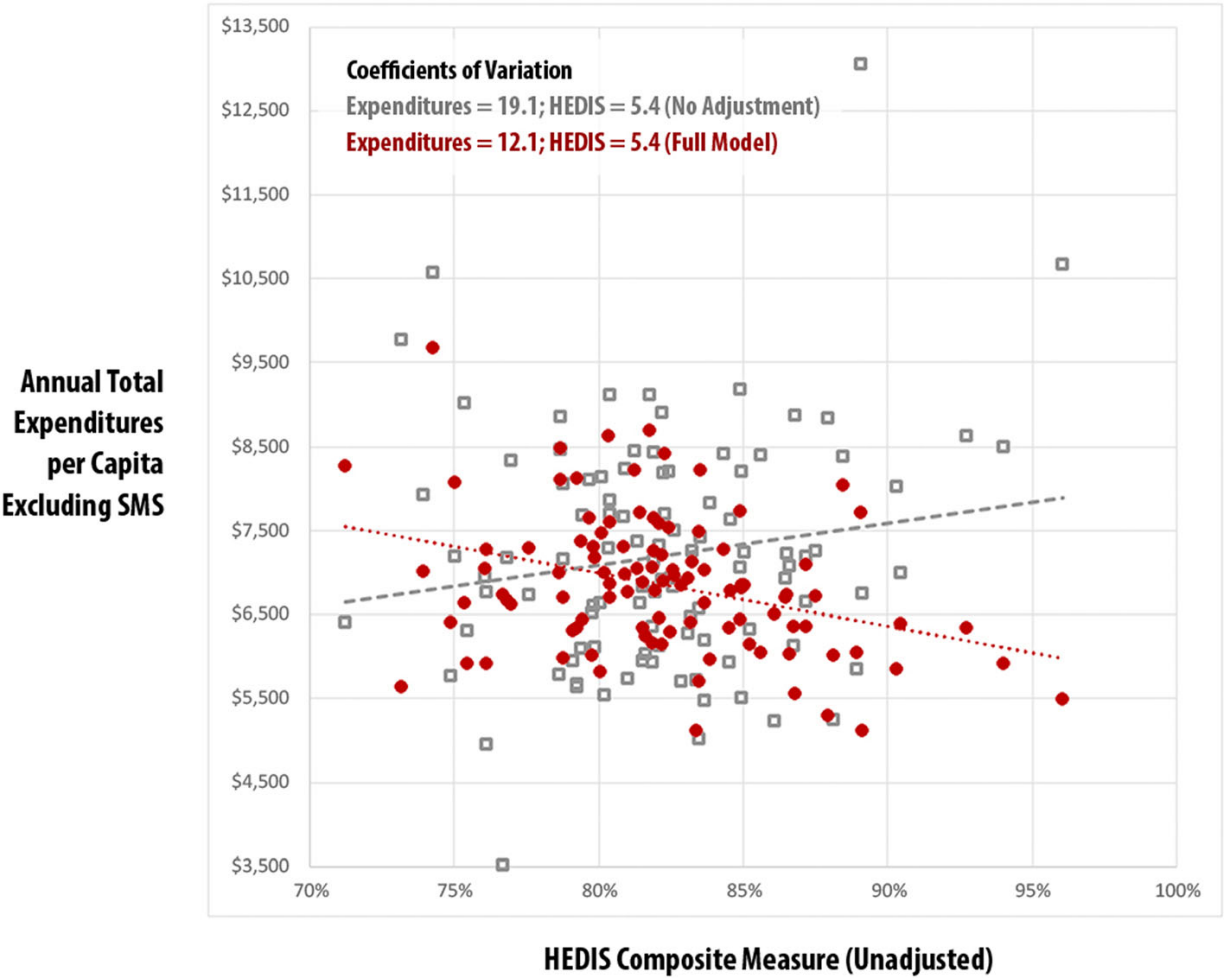
Vermont Blueprint for Health Practice-Level Comparison: Total Expenditures Excluding Special Medicaid Services (SMS) vs. Healthcare Effectiveness and Data Information Set (HEDIS) Composite – Adult, No Adjustment vs. Full Model, CY2014

**Fig. 3:**
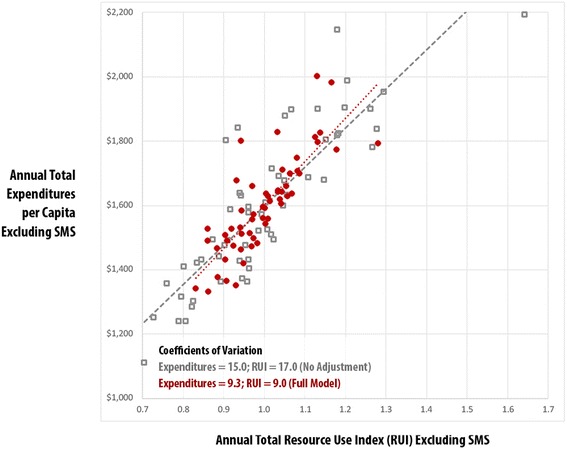
Vermont Blueprint for Health Practice-Level Comparison: Total Expenditures vs. Total Resource Use Index (RUI) Excluding Special Medicaid Services (SMS) – Pediatric, No Adjustment vs. Full Model, CY2014

**Fig. 4:**
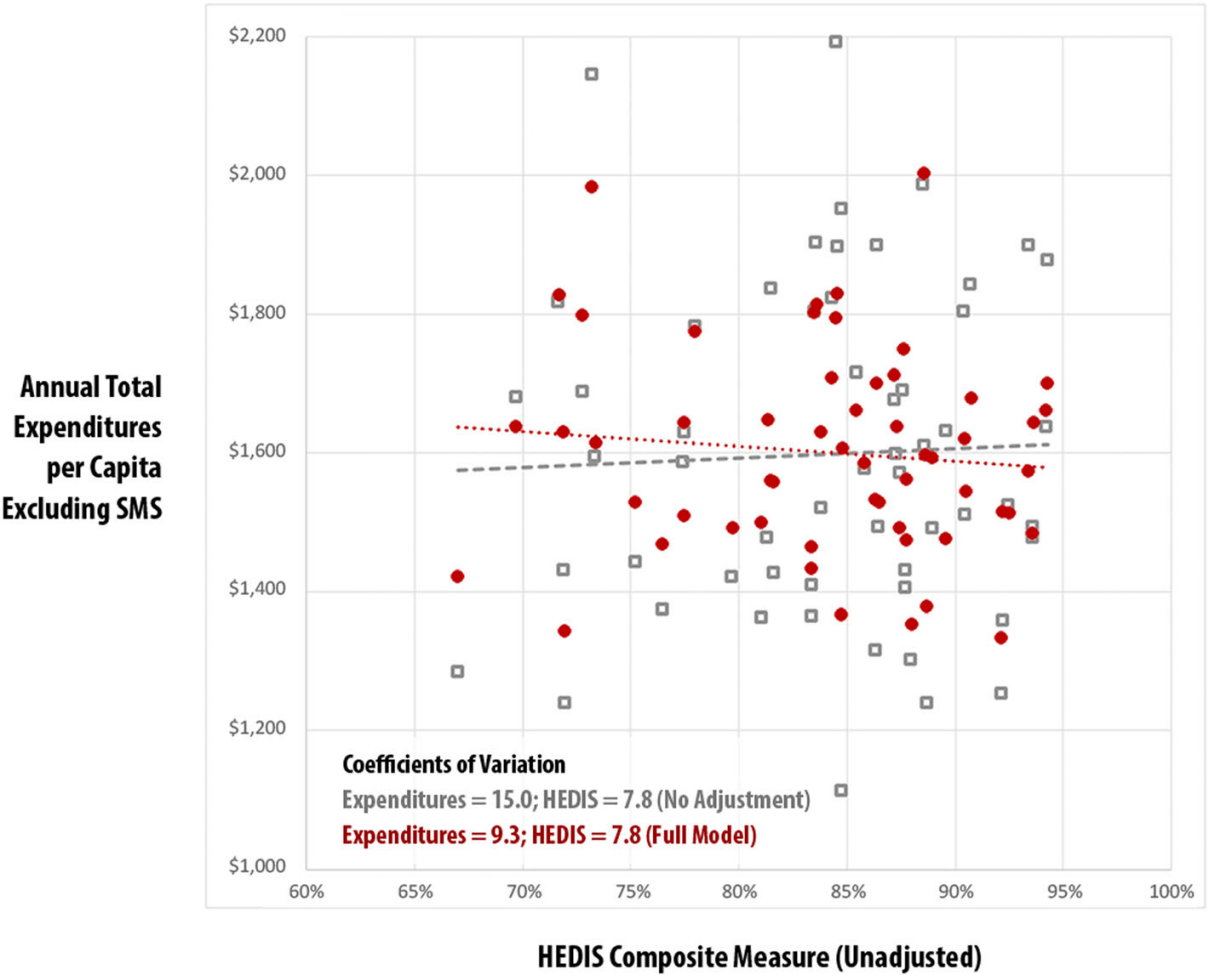
Vermont Blueprint for Health Practice-Level Comparison: Total Expenditures Excluding Special Medicaid Services (SMS) vs. Healthcare Effectiveness and Data Information Set (HEDIS) Composite – Pediatric, No Adjustment vs. Full Model, CY2014

Using the practice-level risk-adjusted expenditures, total Resource Use Index (RUI) rates, and HEDIS composite measures, Figs. 1, 2, 3 and 4 compare practice-level variation between the no-adjustment and full models.

Figure 1 displays the adult population’s practice-level association between total expenditures per capita and total Resource Use Index (both excluding SMS). As measured by the practice-level coefficient of variation, there was less variation in the full model adjustment for expenditures (12.1 vs. 19.1) and RUI (11.5 vs 22.4). The results also indicate that utilization is significantly associated with expenditures at the practice level for the full model (r^2^ .83, *p* < .001).

Figure 2 displays the adult population’s practice-level association between the HEDIS composite measure and Total Expenditures Per Capita Excluding SMS. Because the HEDIS composite is not risk adjusted the coefficient of variation (5.4) was identical for both models. While the no-adjustment model indicated a moderate positive association between HEDIS composite, the full model indicated a negative association between HEDIS composite and expenditures (r^2^ .12, *p* < .001). This underscores the potential for differential interpretation of results when risk-adjustment is applied. In this case, using the full model suggests the possibility that practices with improved higher HEDIS composite score had lower expenditures.

Figures 3 and 4 display no-adjustment and full-model adjustment results at the practice level for the pediatric population. Like the adult population, there was less variation in in the full model than the no adjustment model in the coefficient of variation for expenditures (9.3 vs. 15.0) and RUI (9.0 vs 17.0) and strong association between utilization and expenditures in the full model results (r^2^ .67, *p* < .001). Unlike the adult model, no association between HEDIS composite and expenditures was found in the full model results (r^2^ .01, *p* = .458).

## Discussion

This study assessed risk-adjustment models in support of a “whole-population” approach to measuring and comparing healthcare expenditures and utilization at the practice level. The findings suggest that a more complete risk-adjustment model is appropriate for all-payer, “whole-population” analytics when comparing outcomes at the practice level; this study’s results are in line with a limited number of studies that support the need for risk adjustment. Pope, et al. demonstrated the importance of adjusting for additional variables beyond age and gender for improving the prediction of expenditures [24] However, that study was limited to Medicare data. Another study concluded that risk-adjusted performance measures based on multi-payer claims data were feasible for the assessment of performance and bundled payments to medical homes [25]. Additional studies that applied and compared robust risk-adjustment methods for “whole populations” were not found, indicating a gap that this paper begins to fill.

This risk-adjustment method has been used in the comparative practice profiles developed by the Blueprint, which evaluates practices’ whole populations relative to other practices and to HSA and state outcomes. Following the initial work with state stakeholders on the development of risk-adjustment methods, these profiles, which use the full risk-adjustment model, have been replicated semi-annually for several years, consistently yielding similar results for practices.

The adult and pediatric populations were addressed separately due to the differences in the health status, utilization, and expenditure patterns specific to each population. For example, within the pediatric population, more than half of expenditures for the combined commercial and Medicaid populations were for special Medicaid services (SMS). It should be noted, however, that while this variable was a significant predictor in the Vermont model, coverage of these non-medical social support services may vary by state [26]. Spending on social services and public health as a percent of state gross domestic product (GDP) varies significantly by state, with Vermont ranking highest in the country based on a recent study [27].

Practice-level results indicated that practices varied significantly in patient demographics, health status, and payer mix, so additional payer-specific factors were included in the model to control for these factors. Risk-adjusting for these differences reduced the variability between practices in reported expenditures and total resource use measures. As measured by r^2^ and the log-likelihood ratio test, the predictive ability of the full model over the no-adjustment and simpler models was demonstrated. Measured at the practice level by the coefficient of variation, the reduction in variation between practices using the full model was substantial. For the adult population, a modest practice-level association between higher composite HEDIS preventive and effective care measures and lower per capita expenditures was found for the full-model results but not for the no-adjustment model.

The Blueprint primary care practice profiles are disseminated semi-annually to participating practices. While practice-specific results have not been made publicly available to date, the profiles have provided a tool for the Blueprint program and participating practices to understand expenditure, utilization, and quality of care outcomes relative to other practices in their HSA and across the state. The profiles have contributed to a new level of dialogue and data use across practices and organizations and are routinely used to guide quality and coordination initiatives in each HSA.

This study used a parsimonious model. Additional socioeconomic factors (e.g., housing, income, race, ethnicity, etc.) as well as self-reported health status may improve the model, but were not available in the APCD. A practice’s percent Medicaid served as a proxy for the socioeconomic status of patients in the practice. This paper reports actual payments (i.e., allowed amount) which may be subject to regional variation in prices [28] and a Resource Use Index that is a price-neutral comparison of resource consumption.

A sample of HEDIS measures were selected for this study to create a simple, overall composite measure. The purpose of this study was not to examine a larger sampling of quality measures, but instead to evaluate and demonstrate that there can be a change in the relationship between quality and expenditure measures when risk adjustment is applied to the expenditure measures. Strong correlations between quality measures and expenditure measures also were not found in other studies [29]. Debate continues at the national level as to whether effective and preventive care quality measures should be risk adjusted. Since these measures represent recommended care according to clinical guidelines, the case can be made to not risk-adjust these measures in a “whole-population” system.

This study did not compare or make recommendations regarding health status software or calculations. Rather, it used the commercially available 3M™ CRG system that performs well in identifying conditions associated with cost across the age spectrum. Other studies have compared different health status software systems [10, 18]. All claims-based systems have a limitation when there is a correlation between visits intensity and illness reported — that is, patients with more visits tend to appear “sicker” or have more diagnoses than similar people treated in less intensive environments [30].

This was a quantitative study. Formal qualitative research to use focus groups, interviews, or survey practices to determine their perspectives on what other drivers of differences between high-cost practices versus low-cost practices may prove beneficial.

## Conclusions

This study demonstrated the feasibility of combining populations covered by commercial, Medicaid, and Medicare into a single “whole-population” measurement system. The study demonstrated that primary care practices varied significantly in demographics, health status, payer mix, and patients receiving social support services from Medicaid for their adult and pediatric populations. It also indicated that an enhanced risk-adjustment model improved the predictive power and reduced practice-level variation for total expenditure and utilization measures reporting, which has helped to gain acceptance among providers and other stakeholders in contrast to single-payer reports on practice subpopulations. In light of efforts taking place across the United States to move towards an accountable health system, the “whole-population” measurement approach may have value to accountable care organizations, policymakers, and consumers alike.

## Abbreviations

ACO: Accountable care organization
APCD: All-payer claims database
BCS: Breast cancer screening
CDC: Comprehensive diabetes care
CHF: Congestive heart failure
CMMI: Center for Medicare & Medicaid Innovation
CMS: U.S. Centers For Medicare & Medicaid Services
COPD: Chronic obstructive pulmonary disease
CPC+: Comprehensive Primary Care Plus
CPCi: Comprehensive Primary Care initiative
CRG: 3M Clinical Risk Group
CV: Coefficient of variation
CWP: Appropriate Testing for Children With Pharyngitis (an NCQA HEDIS measure)
E&M: Evaluation and Management
ESRD: End-stage renal disease
GENMOD: Generalized Linear Model Analysis
HCC: Hierarchical Clinical Classification
HEDIS: Healthcare Effectiveness Data and Information Set
HSA: Health service area
IQR: Interquartile range
LBP: Use of Imaging Studies for Low Back Pain (an NCQA HEDIS measure)
MAPCP: Multi-Payer Advanced Primary Care Practice
NCQA: National Committee for Quality Assurance
NQF: National Quality Forum
PCMH: Patient-centered medical home
RUI: Resource Use Index
SAS: Statistical Analysis System
SD: Standard deviation
SIM: State Innovation Model
SMS: Special Medicaid services
TCOC: Total Cost of Care
TCRRV: Total Care Relative Resource Value
URI: Appropriate Treatment for Children with Upper Respiratory Infection (an NCQA HEDIS measure)
VHCURES: Vermont Health Care Uniform Reporting & Evaluation System
